# Supplementary material for: DNA Methylation Signatures of Multiple Sclerosis Occur Independently of Known Genetic Risk and Are Primarily Attributed to B Cells and Monocytes
Source: Int J Mol Sci. 2023 Aug 8;24(16):12576. doi: 10.3390/ijms241612576 (PMC10454485; doi:10.3390/ijms241612576)
Supplement: Supplementary file 1 [file ijms-24-12576-s001.zip › Supplementary figures_and_tables.pdf]

**Supplementary table 1. Group Characteristics.**

| | Discovery | Replication 1 | Replication 2 |
|---|---|---|---|
| Sex | | | |
| Female (N) | 466 | 202 | 337 |
| Male (N) | 141 | 77 | 0 |
| M:F ratio | 0.30 | 0.38 | 0 |
| Group | | | |
| MS Case (N) | 208 | 140 | 235 |
| Non-MS Control (N) | 402 | 139 | 102 |
| Case:Control ratio | 0.52 | 1.01 | 2.30 |
| Age | | | |
| Mean (± SD) | 38.8 (± 9.4) | 41.2 (± 11.2) | 51.8 (± 11.0) |
| Smoker Status (cases only) | | | |
| Ever (N) | 131 | 71 | N/A |
| Never (N) | 75 | 69 | N/A |
| No data | 2 | 0 | N/A |

**Supplementary Table 2: The 13 DMPs at the HLA-DRB locus in all three groups.** All DMPs with **Δβ** > 2% and genome-wide significant in the discovery group as well as FDR ≤ 0.05 and > 2% in both validation cohorts. **Δβ** = delta beta

| CpG | Chr | Position | Gene | Feature | Discovery Cohort Δβ | Discovery Cohort p-value | Validation Cohort 1 Δβ | Validation Cohort 1 p-value | Validation Cohort 2 Δβ | Validation Cohort 2 p-value |
|---|---|---|---|---|---|---|---|---|---|---|
| cg01341801 | 6 | 32489203 | HLA-DRB5 | Body | 0.192 | 3.43E-11 | 0.212 | 6.51E-07 | 0.276 | 1.94E-05 |
| cg25140213 | 6 | 32522683 | HLA-DRB6 | Body | 0.187 | 3.14E-10 | 0.189 | 1.63E-06 | 0.238 | 4.55E-05 |
| cg17369694 | 6 | 32485396 | HLA-DRB5 | 3'UTR | 0.155 | 2.65E-11 | 0.174 | 1.90E-06 | 0.217 | 1.46E-04 |
| cg09139047 | 6 | 32552042 | HLA-DRB1 | Body | -0.135 | 1.36E-08 | -0.162 | 2.70E-06 | -0.175 | 2.70E-03 |
| cg14645244 | 6 | 32552205 | HLA-DRB1 | Body | -0.122 | 7.18E-10 | -0.131 | 6.30E-07 | -0.128 | 2.46E-04 |
| cg10632894 | 6 | 32552453 | HLA-DRB1 | Body | -0.12 | 6.55E-08 | -0.127 | 2.01E-06 | -0.207 | 5.35E-05 |
| cg08845336 | 6 | 32551891 | HLA-DRB1 | Body | -0.119 | 4.01E-09 | -0.14 | 2.16E-06 | -0.137 | 4.94E-05 |
| cg10104420 | 6 | 32526414 | HLA-DRB6 | Body | -0.107 | 1.96E-09 | -0.135 | 5.60E-07 | -0.071 | 3.11E-02 |
| cg19575208 | 6 | 32551888 | HLA-DRB1 | Body | -0.105 | 3.55E-09 | -0.121 | 3.26E-06 | -0.128 | 9.10E-05 |
| cg17416722 | 6 | 32554385 | HLA-DRB1 | Body | 0.096 | 4.67E-11 | 0.105 | 1.88E-06 | 0.14 | 2.72E-04 |
| cg00440797 | 6 | 32493873 | HLA-DRB5 | Body | 0.079 | 5.40E-10 | 0.07 | 5.95E-05 | 0.124 | 1.24E-04 |
| cg00579921 | 6 | 32489991 | HLA-DRB5 | Body | 0.064 | 7.06E-08 | 0.051 | 6.31E-04 | 0.101 | 6.14E-03 |
| cg26036029 | 6 | 32552443 | HLA-DRB1 | Body | -0.061 | 4.72E-08 | -0.042 | 5.30E-05 | -0.076 | 8.69E-05 |
| cg22930808 | 3 | 12228188 | PARP9 | 5'UTR | -0.043 | 6.66E-08 | -0.079 | 8.45E-08 | -0.088 | 5.90E-04 |
| cg01028142 | 2 | 7004578 | CMPK2 | Body | -0.036 | 6.28E-12 | -0.039 | 4.77E-06 | -0.062 | 1.57E-05 |
| cg01533966 | 1 | 90363165 | LRRC8D | 5'UTR | 0.03 | 1.41E-12 | 0.019 | 3.94E-02 | 0.03 | 2.47E-02 |

| | | | | | | | | | | |
|---|---|---|---|---|---|---|---|---|---|---|
| cg01311537 | 10 | 50386709 | TMEM273 | Body | -0.021 | 4.98E-08 | -0.017 | 7.78E-03 | -0.035 | 4.08E-03 |
| cg10778971 | 14 | 94577101 | IFI27 | 5'UTR | -0.02 | 4.43E-12 | -0.014 | 1.15E-03 | -0.014 | 9.12E-03 |

**Supplementary Table 3 – IFN-related DMPs in Ausimmune before and after removal of IFN treated samples**

| CpG | Chromosome | Position | Gene | Only Untreated Δβ | Only Untreated p-value | All Samples Δβ | All Samples p-value |
|---|---|---|---|---|---|---|---|
| cg01028142 | 2 | 7004578 | *CMPK2* | -0.02 | 3.50E-07 | -0.036 | 6.28E-12 |
| cg01533966 | 1 | 90363165 | *LRRC8D* | 0.03 | 2.21E-11 | 0.03 | 1.41E-12 |
| cg10778971 | 14 | 94577101 | *IFI27* | -0.01 | 2.06E-08 | -0.02 | 4.43E-12 |
| cg22930808 | 3 | 122281881 | *PARP9* | -0.03 | 3.26E-04 | -0.043 | 6.66E-08 |

**Supplementary Table 4: DMRs in the combined cohort.**

| Chromosome | Start | End | Width | Number of CpGs | Min Smoothed FDR | Maximum Difference | Mean Difference | Overlapping Genes | DMR Index |
|---|---|---|---|---|---|---|---|---|---|
| chr6 | 32548321 | 32550067 | 1747 | 10 | | 0.161 | 0.051 | HLA-DRB1 | 1 |
| chr6 | 32551749 | 32552670 | 922 | 19 | | -0.167 | -0.077 | HLA-DRB1 | 2 |
| chr6 | 32553920 | 32556093 | 2174 | 5 | | 0.109 | 0.059 | HLA-DRB1 | 3 |

| | | | | | | | | | |
|---|---|---|---|---|---|---|---|---|---|
| chr6 | 32557422 | 32558459 | 1038 | 5 | | 0.126 | 0.029 | HLA-DRB1 | 4 |
| chr6 | 32489203 | 32490444 | 1242 | 12 | | 0.182 | 0.056 | HLA-DRB5 | 5 |
| chr6 | 32520615 | 32523136 | 2522 | 11 | | 0.152 | 0.064 | HLA-DRB6 | 6 |
| chr6 | 32525805 | 32526702 | 898 | 12 | | 0.117 | 0.034 | HLA-DRB6 | 7 |
| chr6 | 32604564 | 32610971 | 6408 | 24 | | 0.113 | 0.043 | HLA-DQA1 | 8 |
| chr6 | 32632000 | 32636189 | 4190 | 40 | | -0.111 | -0.012 | HLA-DQB1 | 9 |

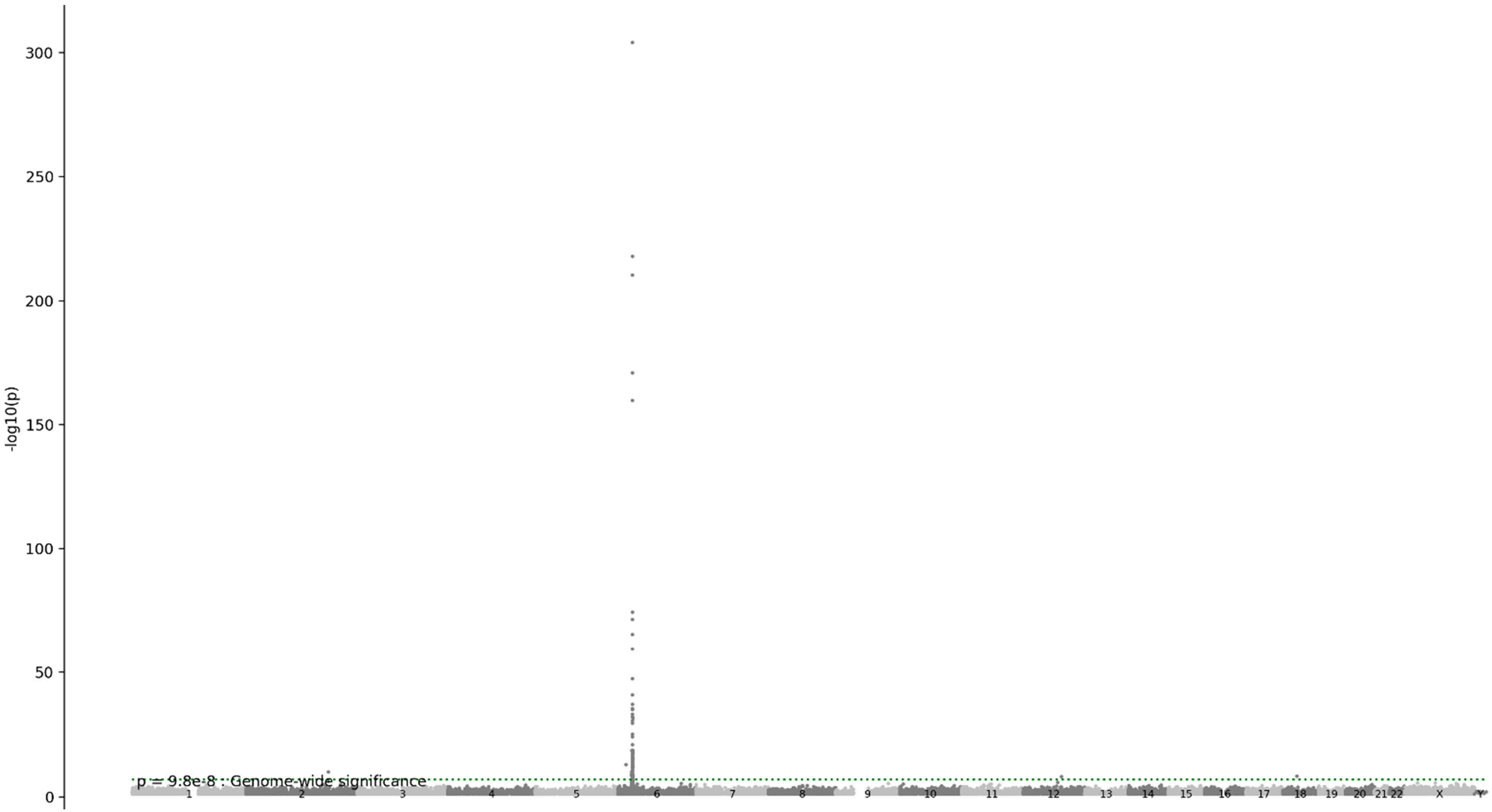


**Supplementary figure 1**: **Manhattan plot showing the magnitude of the *HLA-DRB1 15:01* Haplotype on methylation**. Each dot represents the –log10(p-value) of the ANOVA test between Risk Haplotype HLA-DRB1 15:01 (Presence/Absence) and CpG. Chromosomes are shown with alternating shades of grey)

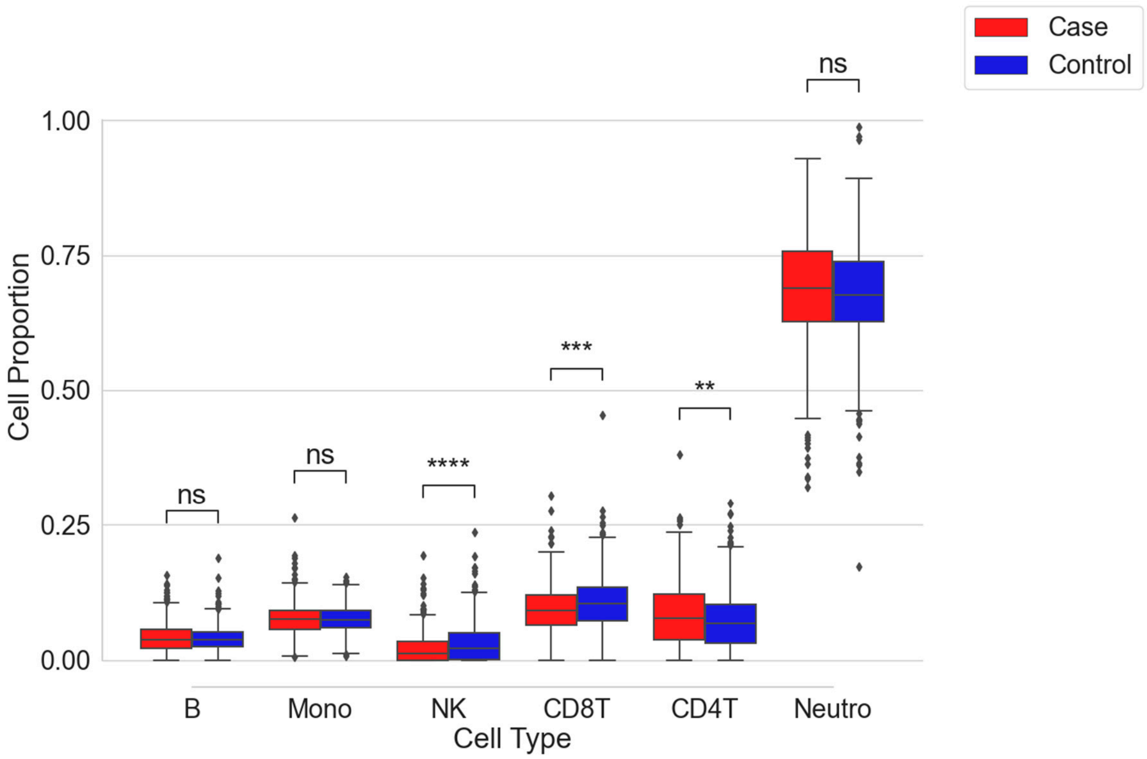


**Supplemental Figure 2: Cell proportion differences between cases and controls using the combined cohort**

Tukey Box Plot showing mean and interquartile range of cases (blue) and controls (orange) in all datasets. Black diamonds indicate outliers. ** P<0.01, ***P<0.001, ****P<0.0001, ns = not significant.

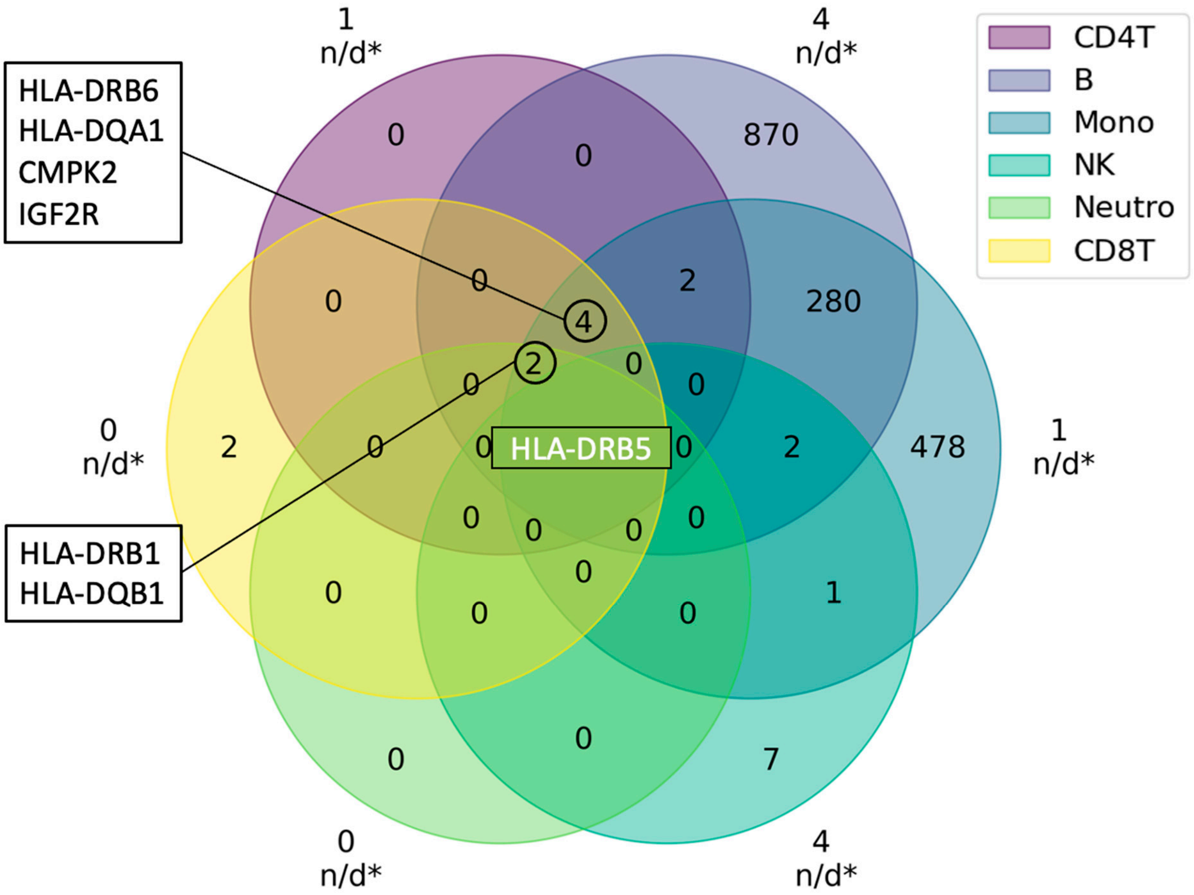


**Supplementary Figure 3. Overlap of DMPs among cell subtypes.** Venn diagram showing overlap of csDMPs in the combined cohort, between all the cell types. *denotes elements of set intersections that are not displayed. For example, only shared between CD4+T cells and NK cells.

**Supplementary Table 5: Overlapping csDMP effect size.** Effect size, chromosome location (CHR) and gene name for the 28 csDMPs which overlap between all 4 major cell types. Red = hypomethylated blue = hypermethylated. Darker shade indicates larger effect size.

| csDMP | B Cell | Mono | $CD8^+$ T Cells | $CD4^+$ T Cells | CHR | Position | gene |
|---|---|---|---|---|---|---|---|
| cg01028142 | -4.70 | -3.61 | -2.06 | -2.65 | 2 | 7004578 | CMPK2 |
| cg10187879 | 4.61 | 2.57 | 2.14 | 2.20 | 6 | 32223208 | |
| cg17369694 | 23.92 | 18.85 | 11.44 | 12.80 | 6 | 32485396 | HLA-DRB5 |
| cg01341801 | 26.79 | 21.29 | 12.76 | 14.76 | 6 | 32489203 | HLA-DRB5 |
| cg26981746 | 12.24 | 9.82 | 5.46 | 6.64 | 6 | 32490012 | HLA-DRB5 |
| cg27362989 | 16.56 | 12.99 | 7.63 | 9.32 | 6 | 32492198 | HLA-DRB5 |
| cg19516921 | 8.63 | 6.97 | 3.90 | 5.11 | 6 | 32493994 | HLA-DRB5 |
| cg19774683 | 18.72 | 14.64 | 8.71 | 10.18 | 6 | 32522400 | HLA-DRB6 |
| cg25140213 | 16.27 | 12.93 | 7.61 | 8.88 | 6 | 32522683 | HLA-DRB6 |
| cg06559318 | 17.40 | 12.78 | 7.29 | 9.57 | 6 | 32526260 | HLA-DRB6 |
| cg10104420 | -11.81 | -8.97 | -5.31 | -5.91 | 6 | 32526414 | HLA-DRB6 |
| cg04422742 | 20.09 | 14.78 | 8.70 | 10.26 | 6 | 32527793 | HLA-DRB6 |
| cg26590106 | 16.53 | 12.43 | 7.58 | 9.28 | 6 | 32548321 | HLA-DRB1 |
| cg12867728 | 4.89 | 2.69 | 2.30 | 2.62 | 6 | 32549283 | HLA-DRB1 |
| cg20022036 | -8.67 | -5.39 | -3.94 | -4.52 | 6 | 32549496 | HLA-DRB1 |
| cg13910785 | 20.98 | 16.20 | 10.21 | 10.69 | 6 | 32549849 | HLA-DRB1 |
| cg11404906 | -19.94 | -14.89 | -9.26 | -10.79 | 6 | 32551749 | HLA-DRB1 |
| cg19575208 | -10.61 | -8.05 | -4.95 | -5.70 | 6 | 32551888 | HLA-DRB1 |
| cg08845336 | -12.48 | -9.78 | -5.94 | -6.93 | 6 | 32551891 | HLA-DRB1 |
| cg15568074 | -22.68 | -16.42 | -9.60 | -11.01 | 6 | 32551949 | HLA-DRB1 |
| cg16514085 | -22.42 | -17.84 | -10.42 | -11.51 | 6 | 32552152 | HLA-DRB1 |
| cg14645244 | -15.93 | -13.15 | -7.17 | -8.67 | 6 | 32552205 | HLA-DRB1 |
| cg10632894 | -16.62 | -13.47 | -7.92 | -9.55 | 6 | 32552453 | HLA-DRB1 |
| cg05383619 | 11.50 | 9.67 | 5.23 | 6.40 | 6 | 32553920 | HLA-DRB1 |
| cg17416722 | 16.44 | 13.60 | 7.80 | 9.03 | 6 | 32554385 | HLA-DRB1 |
| cg02919082 | 8.48 | 6.21 | 3.87 | 4.22 | 6 | 32605694 | HLA-DQA1 |
| cg05341252 | -24.25 | -17.33 | -10.70 | -11.03 | 6 | 32632715 | HLA-DQB1 |
| cg04733681 | 4.81 | 3.41 | 2.21 | 2.53 | 6 | 160423822 | IGF2R |

a)

| | NK | B cell | Monocytes | Neutrophils | CD8T | CD4T |
|---|---|---|---|---|---|---|
| **Total** | 0 | 924 | 1327 | 4 | 475 | 48 |
| **Hypo** | 0 | 517 | 816 | 1 | 361 | 23 |
| **Hyper** | 0 | 407 | 511 | 3 | 114 | 25 |

b)

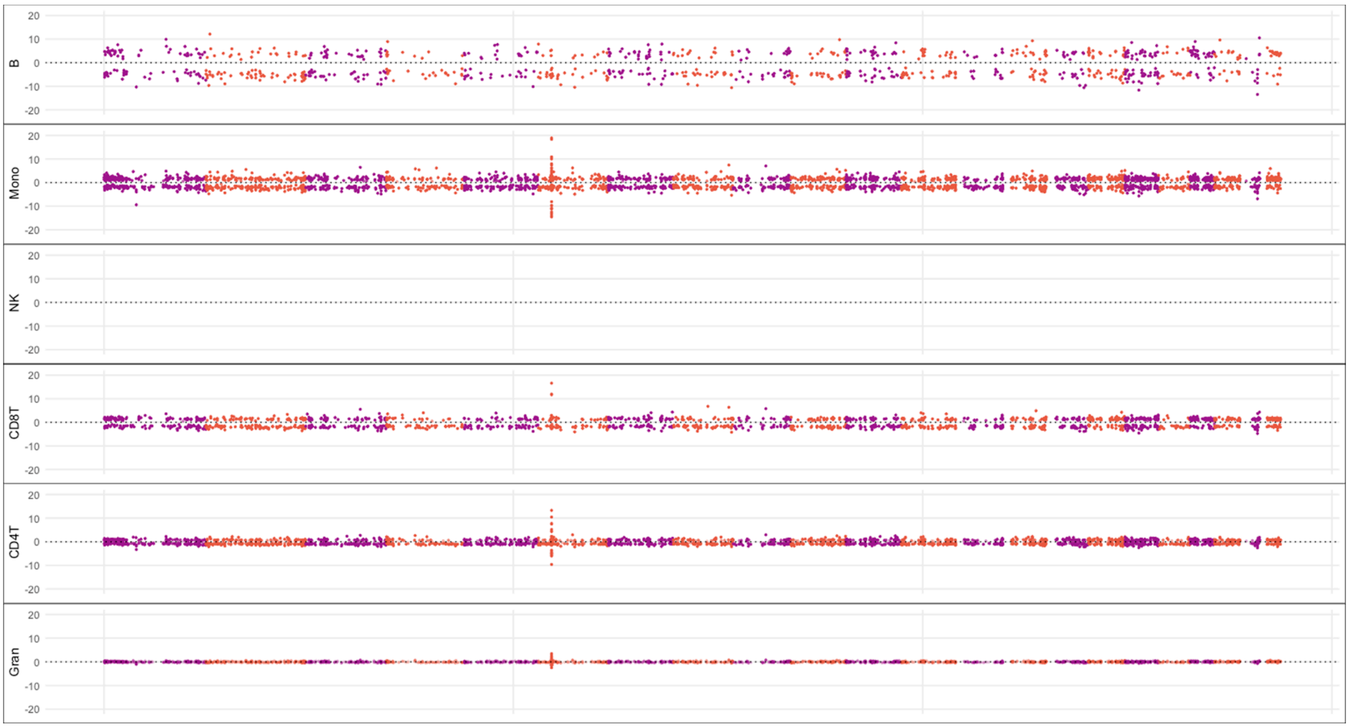


**Supplementary Figure 4: Genome-wide cell-specific DNA methylation profile in discovery group only.**

a) Total number of csDMPs by cell type and breakdown by hyper and hypomethylated. b) Manhattan plot representing genome-wide effect size for all csDMPs, by cell type. The Y axis represents the model estimate for effect size and X axis represents genome-coordinates, chromosomes are in chronological order and represented by alternating colors.

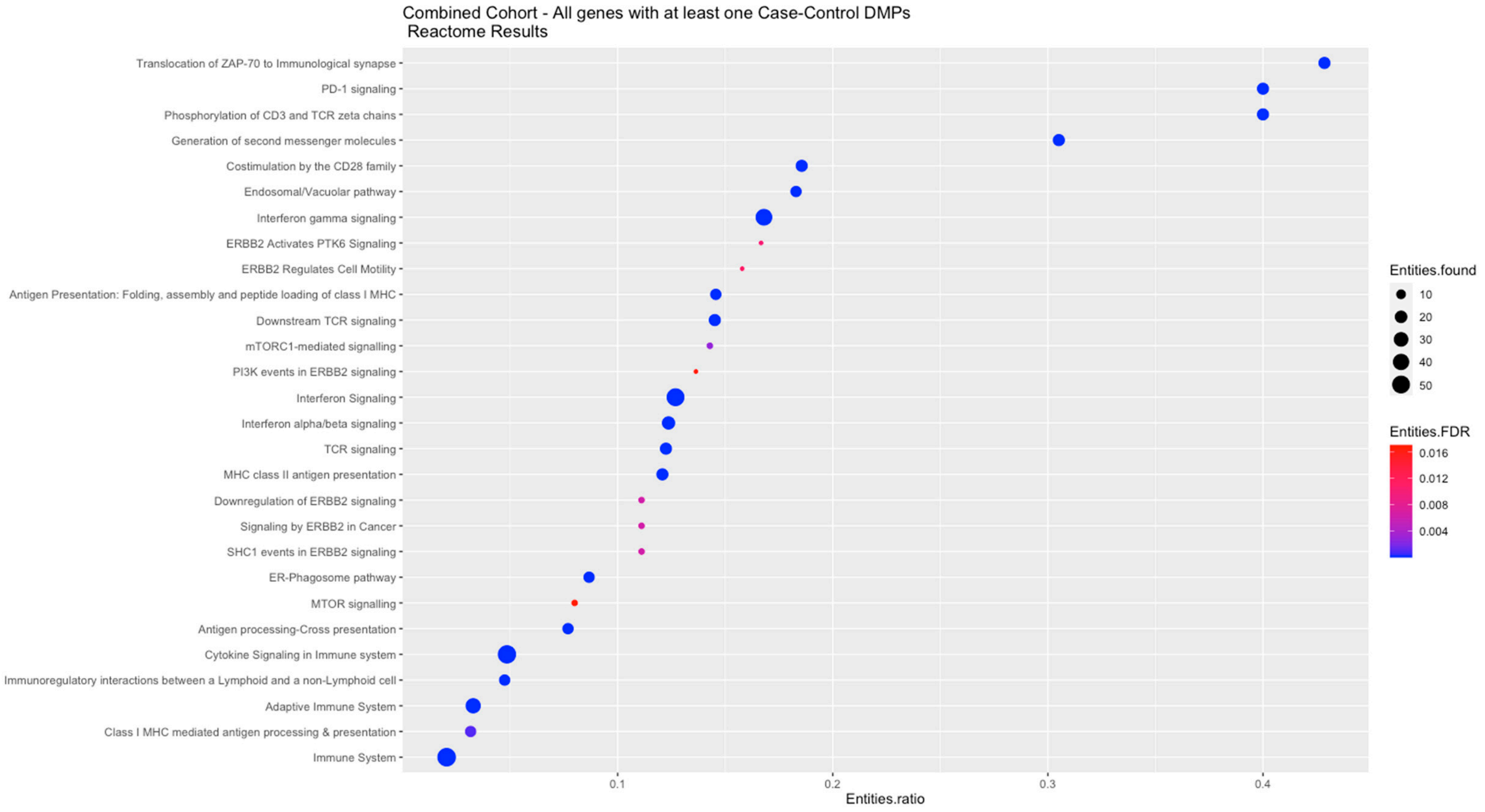


**Supplementary Figure 5: Whole-Blood over-representation analysis**

Using a ranked gene-list with 92 genes containing at least one DMP mapped to a gene with absolute Δβ above 2%. Y axis represents all pathways identified. X axis represents the gene ratio (number of entities in gene list vs number of entities in the pathway) for a specific pathway. Marker size represents the number of Entities. Colour represents significance.

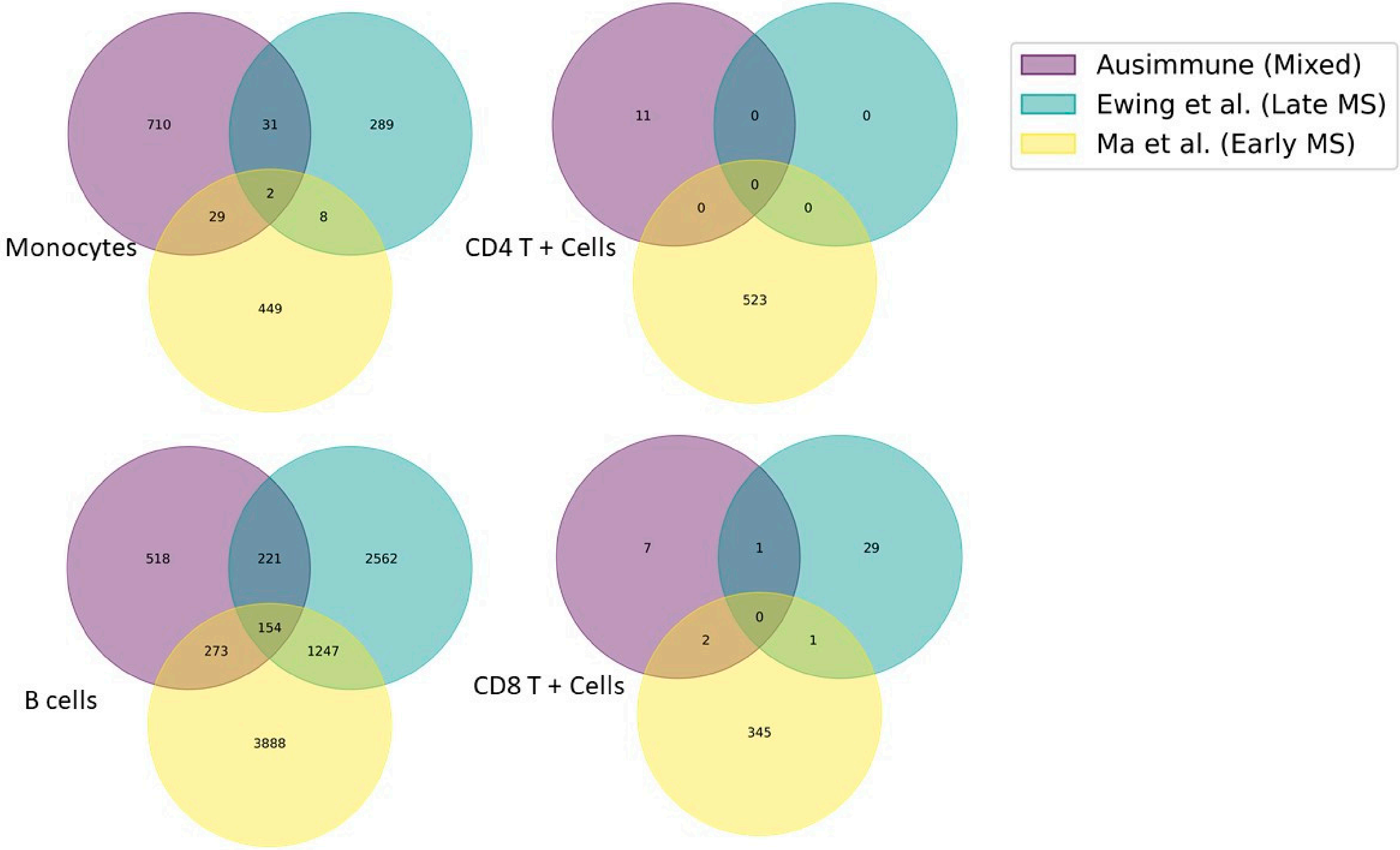


**Supplementary Figure 6: Overlap of Genes containing at least one csDMP identified in this study, Ewing et al. [47] and Ma et al. [33] Study.**
